# A comprehensive laboratory automation system

**DOI:** 10.1155/S1463924685000189

**Published:** 1985

**Authors:** B. H. Venter, M. L. Siebert

**Affiliations:** 1 National Research Institute for Mathematical Sciences NRIMS CSIR PO Box 395 Pretoria 0001 South Africa; 2 National Institute for Water Research NIWR CSIR PO Box 395 Pretoria 0001 South Africa


Journal of Automatic Chemistry/*Journal of Clinical Laboratory Automation*, Vol. 7, No. 2 (Apr–Jun 1985), pp. 80–84

### A comprehensive laboratory automation system

**B. H. Venter**
*National Research Institute for Mathematical Sciences, NRIMS, CSIR, PO Box 395, 0001 Pretoria, South Africa*
**and M. L. Siebert**
*National Institute for Water Research, NIWR, CSIR, PO Box 395, 0001 Pretoria, South Africa*

### Introduction

The computerized system described in this article completely automates the administration and partially automates the operation of the Inorganic Laboratory of the Division of Water Quality of the South African National Institute for Water Research. The purpose of the Laboratory is to perform quantitative inorganic analyses on water samples as a centralized service to the whole Institute, as well as to a number of other research organizations.

The Laboratory receives samples from a large and frequently changing set of sample points throughout South Africa and provides about 80 different determinants, of which researchers may request arbitrary combinations to be performed on any particular sample. Some 120 000 analyses are performed annually. A list of the analytical instruments in use is given in Appendix A.

The analyses are carried out by four staff members, two of whom work only in the morning. Before the introduction of the computerized system, the Laboratory had already implemented an efficient manual system that enabled the staff to cope with the work-load, although it entailed a great deal of paperwork. The computerized system pays for itself mainly by completely eliminating this unproductive and error-prone use of the staff's time. Another major drawback of the manual system was the effort required by the staff to assist researchers with statistical processing of results accumulated over long periods of time.

Before embarking on the in-house development of a computerized system, the Laboratory considered the systems developed by two other local analytical laboratories, a well as proposals from computer vendors. The two laboratory systems, however, were geared to performing a fixed set of analyses on samples from a fixed set of sample points and required reprogramming for every change to the routine. The vendor proposals, on the other hand, were expensive and would have required a complete reorganization of the Laboratory. Consequently, it was decided to develop an in-house system in co-operation with the Computer Science Division of the National Research Institute for Mathematical Sciences.

The aims set for this system were to retain the flexibility, procedures and quality controls of the manual system as far as possible, while:

(1) completely eliminating paperwork and manual calculations by the staff, and
(2) retaining all the results in a permanent data-base and providing a set of programs for the statistical analysis of the results.

A list of the computing equipment used is given in Appendix B. The system has been written in SCRAP, a locally developed systems implementation language, and amounts to about 75 000 lines of source code. The system interacts with its users in Afrikaans, but can produce printed reports in English as and when required.

### System overview

Samples are received by the Laboratory in batches submitted by individual researchers. Each batch is accompanied by a standard form providing the date on which the samples were taken, the names of the sample points, an indication of which determinants are required, and the expected range of the results.The information on the form is immediately entered into the system by a system operator, using a program that is structured around the form. During the entry process, the system assigns a unique number to each of the samples. The numbers are attached to the sample containers by the operator, using pre-printed sticky lables. When all the samples in a batch have been fully analysed, a single report on the batch is returned to the submitter. (An example of such a report is given in figure 2.) When an instrument has been set up to perform an analysis or a set of up to six analyses, for a particular range of values, the operator makes use of a second program that automatically draws up a list of up to 32 samples (identified by their numbers) for which such results still have to be obtained, and whose values are expected to fall within the particular range of results. Although the oldest samples are selected first, the operator may include priority samples in the list.

The samples are then retrieved from storage and a 40-position tray for an automatic sampler is filled in the order specified by the list. Usually, a group of eight samples is preceded by a cup containing a standard solution and a wash cup. The instrument is then started and the computer notified via another program. A real-time executive program then directly monitors the output from the instrument to obtain the results. If necessary, however, the results corresponding to a list of samples may either be entered by the operators via a terminal or digitized from chart recorder output with the aid of a digitizing tablet.

Results obtained directly by the computer are held in temporary storage until an operator runs a program to

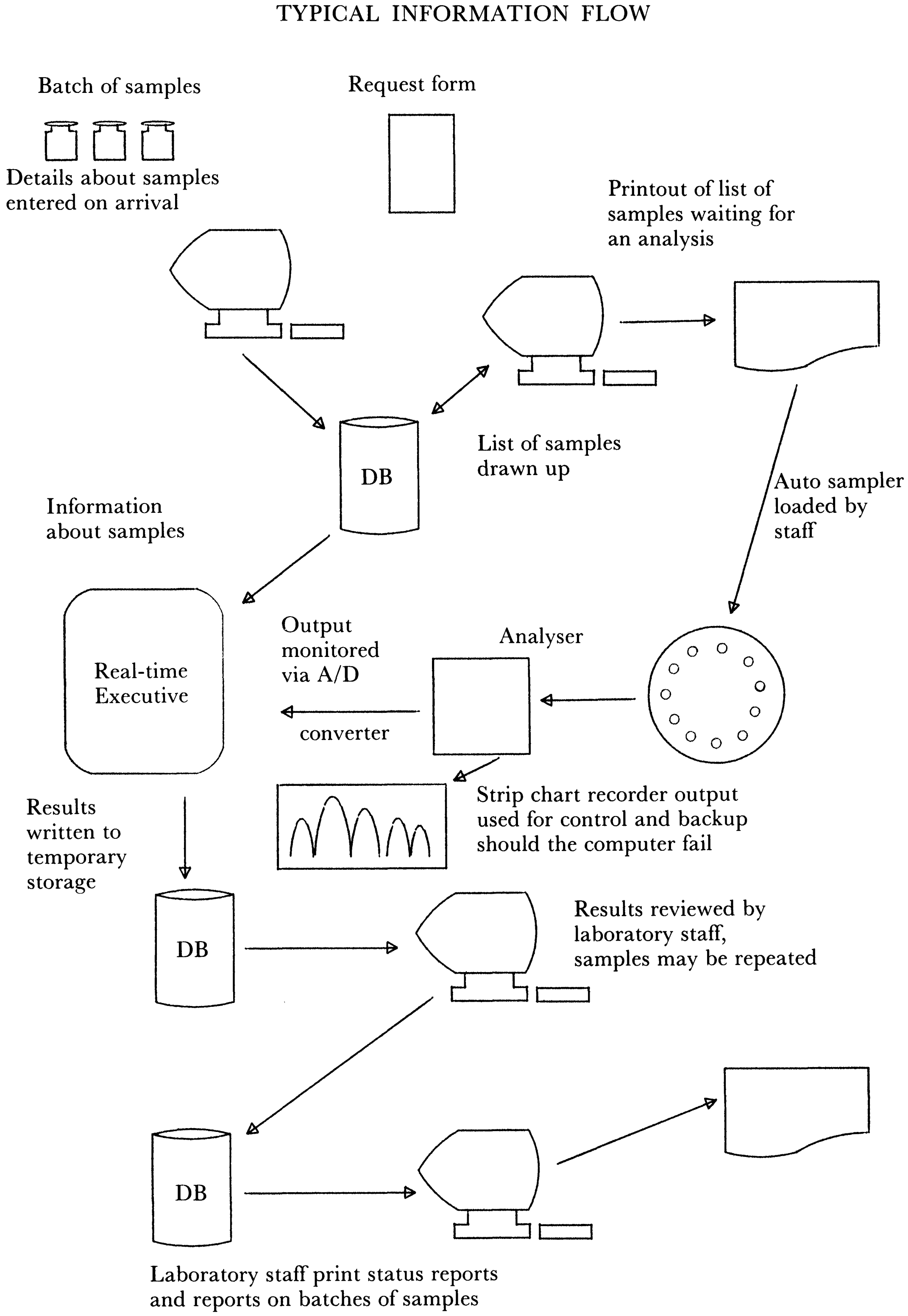

*Figure 1. Typical information flow.*

inspect them for plausibility. Any number of these results can be invalidated by the operator. The samples concerned are then automatically eligible for inclusion in future lists. The system makes extensive provision for abnormal situations and repetition of analyses and can handle samples analysed in diluted form, as well as those with preservatives added.

The status of samples in the system can be monitored continually by the operators by means of a set of reports that can either be printed or displayed on terminals. The most important reports are: a report stating, for each determinant and range of results, how many samples still have to be analysed; a report indicating which batches of samples still have to be reported on (and whether there

SUBMITTER SIEBERT M.L. RESULTS OF CHEMICAL ANALYSES REPORT NUMBER 2791

DATE IN 84-03-23 DATE OUT 84-04-19 84-10-02 DATE SAMPLED 84-03-22 PROJECT NUMBER 1

| | | | | | |
|---|---|---|---|---|---|
| SAMPLE NAME | | RO 163 | RW 206 | RW 207 | SW 64 |
| LABORATORY NUMBER | | 2956 | 2957 | 2958 | 2959 |
| DESCRIPTION | | filtered | filtered | filtered | filtered |
| Corrosiveness | | 0.986 | 19.853 | 3.303 | 0.379 |
| Turbidity (NTU) | | 0.6 | 0.4 | 0.5 | 7.8 |
| pH | | 5.93 | 5.42 | 4.85 | 7.20 |
| Electrical Cond | (mS/m) | 1.04 | 0.87 | 1.27 | 5.95 |
| m Hardness (CaCO3) | | 10.2 | 7.9 | 10.3 | 27.3 |
| m Sum of Salts | | 6.3 | 6.5 | 7.6 | 33.0 |
| m Colour | (mg/l Pt) | <5 | <5 | 7 | 167 |
| m Sodium | (Na) | <1 | <1 | <1 | 2 |
| m Potassium | (K) | <1 | <1 | <1 | <1 |
| m Calcium | (Ca) | 1 | 1 | <1 | 7 |
| m Magnesium | (Mg) | 2 | 1 | 2 | 2 |
| m Silica | (Si) | <0.2 | <0.2 | <0.2 | 1.4 |
| m Sulphate | (SO-4) | <2 | <2 | 2 | <2 |
| m Chloride | (Cl) | <2 | <2 | <2 | 4 |
| m Total alkalinity | (CaCO3) | <2 | <2 | <2 | 17 |
| m Chemical oxygen demand | | 9 | 12 | <5 | 20 |
| m Dissolved Organic carbon | | 2.6 | 2.9 | 1.1 | 6.0 |
| u Kjeldahl nitrogen | (N) | 50 | 135 | 175 | 173 |
| u Ammonia nitrogen | (N) | 24 | 112 | 125 | 37 |
| u Nitrate + Nitrite | (N) | 143 | 142 | 159 | 487 |
| u Nitrite nitrogen | (N) | 6 | 5 | 5 | 11 |
| u Total phosphate | (P) | 52 | 33 | 29 | 276 |
| u Orthophosphate | (P) | 15 | 26 | 28 | 129 |
| u MBAS | (LAS) | 55 | 44 | <25 | 156 |
| u Aluminium | (Al) | <20 | 47 | 39 | 149 |
| u Boron | (B) | 55 | 39 | 73 | 64 |
| u Lead | (Pb) | <25 | <25 | <25 | 28 |
| u Manganese | (Mn) | <25 | <25 | <25 | <25 |
| u Zinc | (Zn) | 34 | 36 | 38 | <25 |
| u Iron | (Fe) | <25 | <25 | <25 | 704 |
| CHEMICAL BALANCE | | balances | balances | balances | balances |

m = MILLIGRAM PER LITRE
u = MICROGRAM PER LITRE

*Figure 2.*

are any results outstanding); and a report detailing the lists of samples that have already been drawn up, but for which results have not yet been read in or have not yet been inspected. The system also produces monthly and yearly reports that summarize the work-load.

A report on a batch of samples may be printed at any time after receipt. All batches are uniquely numbered and permanently accessible. Results that still have to be obtained are indicated as such on the report, which can also contain messages detailing abnormalities encountered. Finally, each report contains the date on which the samples were taken, the date on which the samples were received by the Laboratory, and the date on which the report was sent to the submitter. These dates clearly identify delays and provide motivation for improved laboratory throughput.

A schematic representation of the system information flow is given in figure 1.

### System quality-assurance and safeguards

As a quality-assurance measure, a cup containing a standard solution and followed by a wash cup, is usually inserted before every set of eight samples. The results obtained using the standard solutions are accumulated for each determinant. After 30 such values have been accumulated for a determinant, their relative variance is calculated and the accumulation fields are reset to zero. The values so obtained can be printed-out on demand and give an indication of the accuracy of the analyses. The system also makes provision for processing the results obtained from repeatability tests carried out from time to time on natural samples. The results on the printed reports returned to the submitters include no more digits after the decimal point than is deemed significant for each determinant.

The system is designed to recover from hardware and software failures without losing any information entered prior to the failure. Furthermore, because the system can accept input by means of a digitizing tablet or keyboard entry, the Laboratory can revert to the manual system should the computerized system become inoperative for an extended period of time. When computerized operation resumes, the system can be brought up-to-date and normal operations can be continued, quickly and easily. To ensure recovery from destruction or corruption of the information held on the system's disk drive, the disk is dumped to magnetic tapes daily, and these tapes are placed in remote safe-keeping weekly.

Access to the system is physically restricted to the Laboratory's staff, as well as being password-controlled. In addition, authorized users of the system are given a level of access specifically tailored to their needs and level of responsibility. As a final security and quality-control measure, each report sent out from the Laboratory must

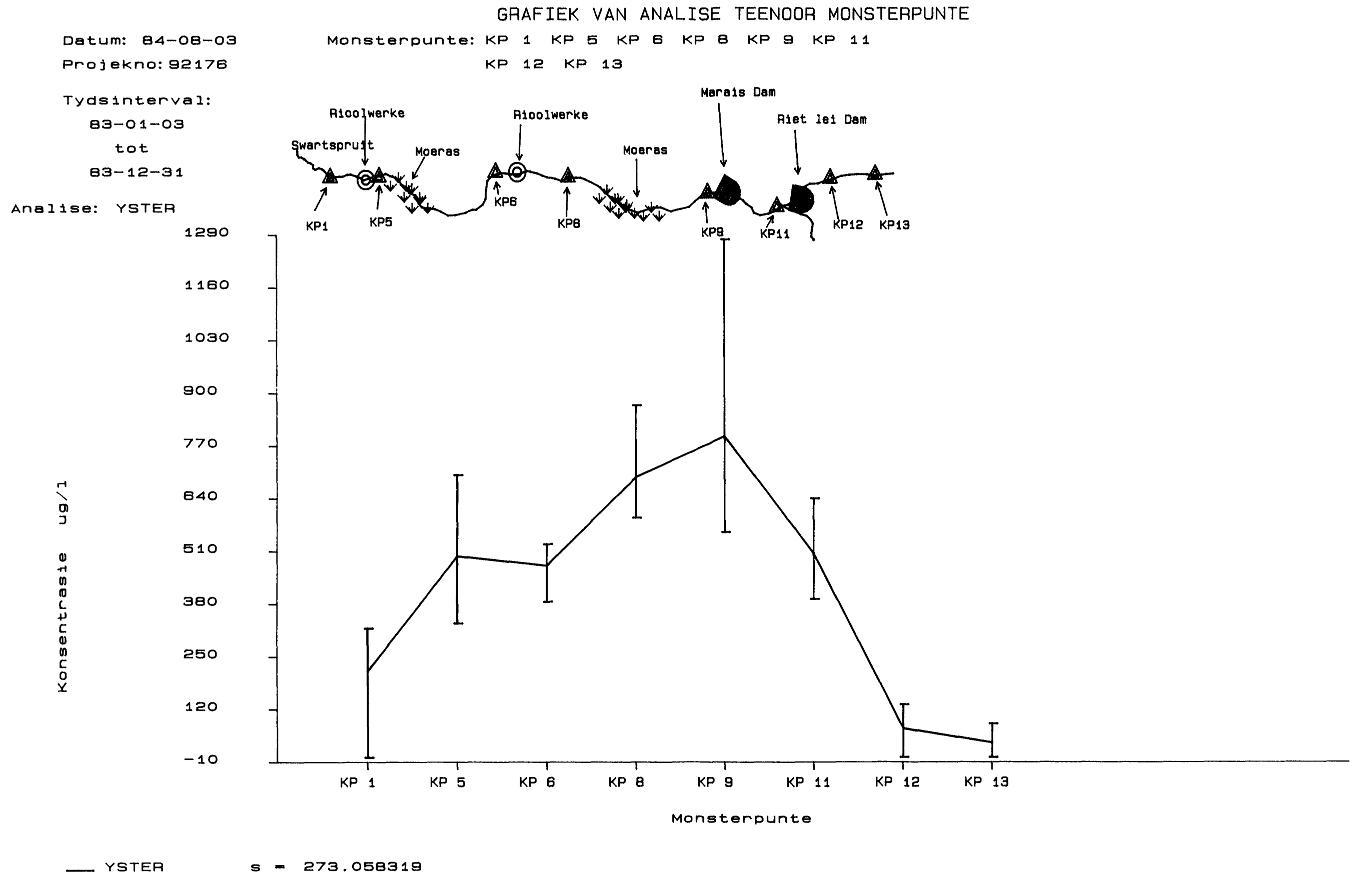

*Figure 3.*

be signed by an authorized operator, and the system instructed that the report is now final and unalterable.

### Statistical processing of results

All results obtained by the Laboratory are stored permanently by the system. This is achieved by systematically copying the results onto magnetic tapes and reusing the space occupied by the oldest results for new results. When needed, selected portions of the archived results can be brought back to the disk storage for use in statistical queries. At the present rate of creating new results, reuse of space will only begin in about 10 years' time.

The stored results may be processed in a number of dfferent ways, the most frequent being to print all the results obtained on samples from a specific sample point during a given period. The results may also be summarized in various graphical formats. An example of a typical graph is shown in figure 3. The map appearing on the graph was fed into the system by means of the digitizing tablet. The operators can create any number of such maps and can use a particular map in any kind of graph.

The results stored in the system can also be placed onto magnetic tapes and used as input to other specialized information systems.

### Experience with the system and conclusions

Since the Laboratory was able to cope with its work-load before the introduction of the computerized system, there has not been any dramatic increase in output. However, the Laboratory's staff now have about 40% more time available for non-routine work and should be able to cope easily with anticipated future increases in the workload. In addition, clerical errors are now fairly rare, and the Laboratory provides a much better service to its clients.

The system is menu driven, and particular emphasis has been placed on minimizing the number of times that the operators have to interact with the system and the number of keystrokes required per interaction. The system proved to be sufficiently friendly for the Laboratory's staff to operate it without the benefit of formal training and long before the availability of a user's manual. Although the system functions automatically to a high degree, the operators are in full control of the system at all times and have been able to cope with all the problems and exceptional situations encountered thus far without having to resort to handling such problems outside of the system.

In the opinion of the authors, much of the success of the system is due to the fact that there was close co-operation between the designers and users of the system during the design phase, with the first author contributing his knowledge of computer systems and the second his knowledge of the Laboratory's needs. A further important factor was the fact that the system was implemented in the Laboratory on the computer used to run the system. Also, parts of the system were already being used, while other parts were still being developed. This exposed the designers to user feedback for over a year and enabled the original specification of the system to converge towards the actual needs of the Laboratory.

Although every effort should be made to set up the original specifications as accurately and completely as possible, a system of this size can never be completely specified in advance before experience of the actual use of the system. Designers of similar systems would be well advised to make every effort to build changeability into their systems.

The system described here is fairly open-ended and, in principle, can cope with any analysis that quantifies a sample into a single number. The hardware can monitor up to 32 analyses simultaneously and, in principle, can be interfaced to any type of analytical instrument.

The entire project involved six people, including two recently graduated trainee programmers, working part-time over a period of 18 months. About five man years were spent.

### Appendix A

*Analytical instruments used by the laboratory*

BECKMAN Total Organic Carbon Analyser
NIWR Dissolved Organic Carbon Analyser
NIWR Total Organo-Halogen Analyser
RADIOMETER CMD 83 Conductivity Meter
TECHNICON Auto-Analyzer II + CSM 6 (16 channels)
VARIAN Techtron AA5 Atomic Absorption Spectrometer
VARIAN 1275 Atomic Absorbtion Spectrometer

### Appendix B

*Computing equipment*

C. ITOH Model 1541R, Dot matrix printer
CENTRONICS 704, Dot matrix printer
HEWLETT-PACKARD 2621B, Visual Display Terminal (three terminals)
HEWLETT-PACKARD 2627A, Colour Graphics Terminal
HEWLETT-PACKARD 7220T, Colour Plotter
PERKIN-ELMER 3210, 32-bit Mini Computer (1 Megabyte)
PERKIN-ELMER 80 Megabyte Fixed Head Disk Drive
PERKIN-ELMER 1600-Bytes per inch Magnetic Tape Drive
SUMMAGRAPHICS Bit Pad One, Digitizing Tablet

### CLINICAL CHEMISTRY AND ENZYMOLOGY MEETINGS – ISRAEL, 1985

Three independent conferences are to be held in Israel this year centred around the Sixth European Congress of Clinical Chemistry (1–5 September, Jerusalem) – the Second Congress of the International Society of Animal Clinical Biochemistry (2–6 September, Jerusalem); the Fifth International Congress on Clinical Enzymology (3–6 September, Jerusalem); and the Fifth International Meeting on Clinical Laboratory Organization and Management (27–29 August, Haifa). The latter includes papers on Chronobiology, Serial laboratory data, Imaging and computer graphics, Laboratory data in disease modelling, Economic challenges to the clinical laboratory:

New technology and economic reality in the clinical laboratory
*B. Statland* (USA)

Applications of economic analysis to the clinical laboratory
*D. Hardwick* (Canada)

System analysis of workflow
*P. Winkel* (Denmark)

Computer-aided analysis in the small clinical laboratory
*P. Bonini* (Italy)

Cost model for quality control
*P. Hyltoft-Peterson* (Denmark)

The economy of clinical laboratory management
*H. Fineberg* (USA)

The cost and benefits of profiling
*P. Broughton* (UK)

Appropriateness of clinical laboratory tests
*J. Buttner* (FR Germany)

The clinical laboratory as a process model
*R. Edwards* (Australia)

and laboratory bedside testing:

Advantages and disadvantages of bedside testing
*V. Marks* (UK)

The laboratory as a clinical entity – interactions and feedback
*O. Zinder* (Israel)

The physician's vantage point in bedside testing
*E. Cerasi* (Israel)

New technologies in bedside testing.

*More information from Dr E. Shafrir, Chairman of the Co-ordinating Board: Clinical Chemistry and Enzymology Meetings, PO Box 50006, Tel-Aviv, Israel. Tel. 03 654571.*

